# Mutation-Driven Signals of ARID1A and PI3K Pathways in Ovarian Carcinomas: Alteration Is An Opportunity

**DOI:** 10.3390/ijms20225732

**Published:** 2019-11-15

**Authors:** Pradip De, Nandini Dey

**Affiliations:** 1 Translational Oncology Laboratory, Avera Cancer Institute, Sioux Falls, SD 57105, USA; Pradip.De@avera.org; 2 Department of Internal Medicine, SSOM, University of South Dakota, Sioux Falls, SD 57105, USA; 3 VieCure, Greenwood Village, CO 80112, USA

**Keywords:** ARID1A, ovarian cancers, cellular signaling, PI3K pathway

## Abstract

The chromosome is a functionally dynamic structure. The dynamic nature of chromosome functionally connects it to almost every event within a cell, in health and sickness. Chromatin remodeling system acts in unison with the cell survival pathway in mediating a variety of cellular functions, including mitosis, differentiation, DNA damage repair, and apoptosis. In humans, the 16 SWI/SNF complexes are a class of nucleosome remodelers, and ARID1A, an epigenetic tumor suppressor, is a member of mammalian 17 chromatin remodeling complex, SWI/SNF. Alterations of chromatin remodeling system contribute to tumorigenic events in various cancers, including ovarian cancers. Oncogenic changes of genes of the PI3K pathway are one of the potential genetic determinants of ovarian carcinomas. In this review, we present the data demonstrating the co-occurrence of mutations of ARID1A and the PI3K pathway in our cohort of ovarian cancers from the Avera Cancer Institute (SD, USA). Taking into account data from our cohort and the cBioPortal, we interrogate the opportunity provided by this co-occurrence in the context of mutation-driven signals in the life cycle of a tumor cell and its response to the targeted anti-tumor drugs.

## 1. Prologue

A eukaryotic chromosome is the best known decisively-packaged product of evolution. The marvel of the packaging of chromosomal DNA by nucleosomes not only condenses the genetic material but also organizes the genome, its millions of years’ worth of evolutionary facts in a millionth of a length. The packaging is at the cost of limiting its access to critical regulatory DNA element(s) meant for the function of the chromosome. And yet a eukaryotic chromosome is dynamic. It is dynamic in its precise transcriptional regulation of a particular gene in a spatiotemporal manner. Years of evolution have enabled a eukaryotic cell to develop controlled dynamic access to the packaged-DNA as well as to tailor its nucleosome composition at a chosen chromosomal region; a regulated alteration of chromatin structure by a set of specialized complexes called remodeling complexes. The chromatin remodeling causes a change in chromatin structure that occur during regulatory processes in a life-cycle of a cell by altering the nuclease sensitivity of a region of chromatin [1]. Remodeling is a covalent modification of histones or an ATP-dependent self-regulated process with the unique property of dynamic repositioning of nucleosomes directed towards a specific biological event, physiological as well as pathological. The chromatin remodeling serves the penultimate purpose of genetic-decoding in a cell to actively participate in the regulation of transcription, DNA replication, DNA damage repair, DNA methylation, recombination, and chromosome segregation during its life-cycle. Precise control of chromatin architecture and its mode of remodeling are vital to all physiological processes as well as pathological transformations as genetic-code packaged in the nucleus are only revealed following chromatin remodeling [2]. By virtue of chromatin remodelers, human chromatin is the most dynamically remodeled structure within a cell, rearranged both spatially and temporally, in health and sickness.

## 2. ARID1A, A Member of the Mammalian Chromatin Remodeling Complex, SWI/SNF

In humans, the SWItch/Sucrose Non-Fermenting (SWI/SNF) complexes, caused by defects in the transcription of the SUC2 invertase gene, are a class of nucleosome remodelers [3]. As one of the members of mammalian chromatin remodeling complexes, the SWI/SNF complex repositions, ejects, or exchanges nucleosomes, which modulate DNA accessibility to cellular processes dependent on chromatin, such as transcription, DNA replication, and DNA repair [4,5,6]. SWI/SNF, essential for activation or inhibition of transcription and thus the expression of many genes, has a stronger affinity for binding to DNA than nucleosomes which has been known to explain the profound structural changes (SWI/SNF creates a more accessible DNA path by displacing the histones) [1,7]. ARID1A (the AT-rich interacting domain-containing protein 1A gene) gene is located on chromosome 1p36.11 and is a core component of the mammalian SWI/SNF complex [8]. ARID1A contains an ARID domain, which interacts with DNA in a sequence-nonspecific manner regulating cellular processes, including proliferation and differentiation, and hence reported to be involved in tumorigenesis [9,10,11]. 

## 3. ARID1A as an Epigenetic Tumor Suppressor

ARID1A ranks top among the mutated chromatin regulator across all human cancers. SWI/SNF is the most frequently mutated chromatin-regulatory complex in human cancers, exhibiting a broad mutation pattern, similar to that of TP53 [12]. Somatic inactivating mutations of ARID1A have been reported to impart aberrant chromatin remodeling in a variety of neoplasms [13], predominantly gynecological cancers. The mutation of ARID1A has been most prevalent in cancers of ovary and endometrium [14]. The mutational status (inactivating) of ARID1A in a broad spectrum of human cancers, including ovarian cancers, established the role of ARID1A as an epigenetic tumor suppressor [15].

## 4. ARID1A Mutation in Ovarian Carcinomas

The mutation of ARID1A has been reported in rare epithelial ovarian tumors, which was first published in 2010 [14,16]. Oncogenic loss of somatic ARID1A protein expression has been identified as an early molecular event during a transformation of endometriosis into cancer (from endometriosis to endometriosis-associated carcinoma in ovarian cancer and also from atypical endometrial hyperplasia to endometrioid adenocarcinoma in endometrial cancer). Also, the loss of ARID1A-encoded protein BAF250a (a subunit of the Brahma-associated factor [BAF] nucleosome remodeling complex) was recorded as a frequent event in both ovarian clear cell and endometrioid carcinomas or in endometriosis-associated ovarian carcinomas [14,17,18]. Although the loss of BAF250a was not associated with clinical or epidemiologic characteristics and there was no relationship between the loss of BAF250a and stage, grade, survival, or epidemiological variables, the loss of ARID1A function as shown by loss of protein expression has been reported as an early molecular event in the development of most ovarian clear cell and endometrioid carcinomas arising in endometriomas [17,19]. Identification of somatic loss of expression of ARID1A protein is aligned to the fact that endometrioid and clear cell ovarian carcinomas might arise through a malignant transformation of endometriotic lesions as postulated by identifying the common genetic changes between endometriosis and ovarian cancers [18,20,21].

**Figure 1:**
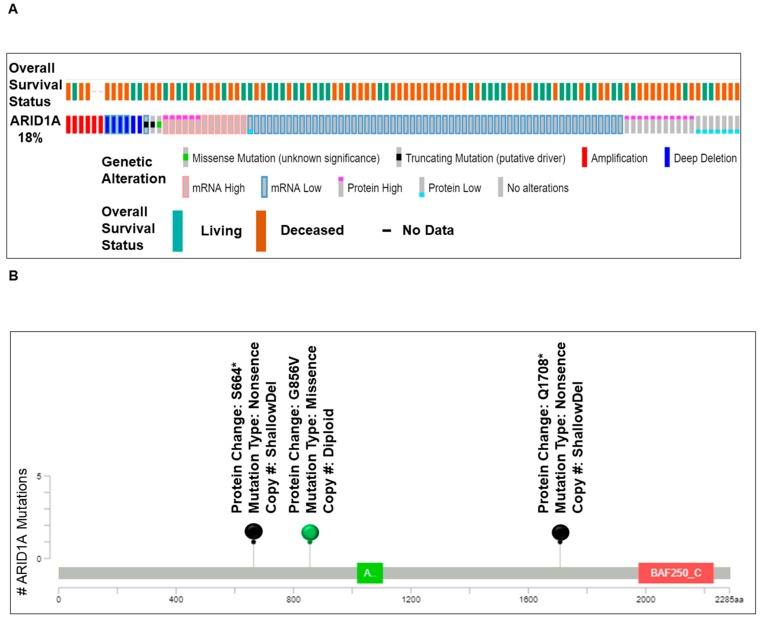

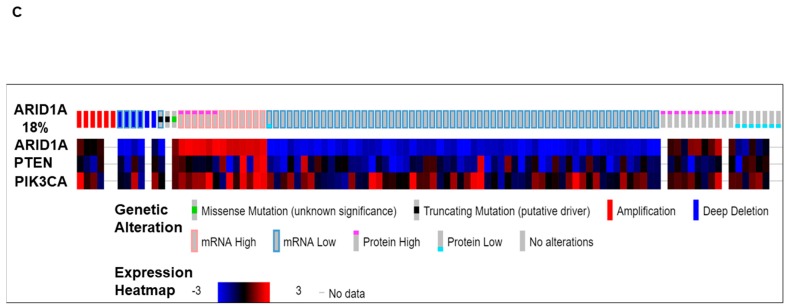
Alterations of ARID1A gene in ovarian cancers. Data has been quarried from cBioPortal (April 2019) representing a study (TCGA Provisional) of 594 patients/606 samples, (http://www.cbioportal.org). Quarried gene is altered in 104 (18%) of queried patients [104 (17%) of queried samples]. (**A**) presents Oncoprint showing the alteration frequency of the ARID1A gene in ovarian serous cystadenocarcinoma (TCGA Provisional) with the overall survival status. The color codes for the types of genetic alterations (missense mutation/truncating mutation/amplification/deep deletion/mRNA high/mRNA low/protein high/protein low/no alterations) are presented in the figure. The overall survival status (living, deceased, and no data) is presented as the inset in the figure. (**B**) presents the number of ARID1A mutations (somatic mutation frequency) (Y-axis) plotted on the amino acid (sequence number as X-axis). Protein Changes, Mutation Types, and Copy numbers are presented in the figure. Mutation diagram circles are color-coded with respect to the corresponding mutation types. The green circle represents missense mutations. Black circles represent truncating mutations (Nonsense, Nonstop, Frameshift deletion, Frameshift insertion, and Splice site). (**C**) presents the expression heatmap of ARID1A, PTEN, and PIK3CA in genes in ovarian serous cystadenocarcinoma (TCGA Provisional) representing a study of 594 patients/606 samples, (http://www.cbioportal.org). The heatmap was superimposed on the alterations of the ARID1A gene in respective samples. We acknowledge works of Cerami et al. [23] and Gao et al. [24]. We acknowledge the TCGA Research Network for generating TCGA datasets.

We present (Figure 1) alterations of the ARID1A gene in tumors from patients with ovarian cancers. Data have been queried from cBioPortal (April 2019), representing a study (TCGA Provisional) of 594 patients/606 samples, (http://www.cbioportal.org). Quarried gene was altered in 104 (18%) of queried patients [104 (17%) of queried samples]. Figure 1A presents Oncoprint showing the alteration frequency of the ARID1A gene in ovarian serous cystadenocarcinoma (TCGA Provisional) with the overall survival status. The color codes for the types of genetic alterations (missense mutation/truncating mutation/amplification/deep deletion/mRNA high/mRNA low/protein high/protein low/no alterations) are presented in the figure. The overall survival status (living, deceased, and no data) is presented as the inset in the figure. A recent study from Heckl et al. demonstrated that ARID1A, along with p53 and beta-catenin is the strongest prognostic factor of tumor suppressor genes in clear cell and endometrioid subtypes of ovarian and endometrial cancers. The survival analysis in their study showed that negative expression of ARID1A is one of the three independent negative prognosticators in both clear cell and endometrioid carcinoma, regardless of ovarian or uterine origin. Cox-Regression analysis confirmed ARID1A as negative prognostic factors [22]. Figure 1B shows the relative distribution of the types of mutations of ARID1A observed in ovarian patients. The number of ARID1A mutations (somatic mutation frequency) (Y-axis) is plotted on the amino acid (sequence number as X-axis). “Protein Changes,” “Mutation Types,” and “Copy Numbers” are presented in the figure indicating specific sites where the protein(s) is altered, S664*, G856V, and Q1708*. Mutation types are either non-sense or missense, while the copy numbers varied from shallow deletion to diploid. Figure 1C presents the expression heatmap of ARID1A, and two genes of the PI3K pathway, PTEN and PIK3CA in ovarian serous cystadenocarcinoma (TCGA Provisional) representing a study of 594 patients/606 samples, (http://www.cbioportal.org). The heatmap was superimposed on the alterations of the ARID1A gene in respective samples. In patients with a low expression of mRNA/protein for ARID1A, alterations of PTEN and PIK3CA were observed.

## 5. Alterations in the ARID1A Gene and Its Co-Occurrence with Alterations of the PI3K Pathway Genes in Ovarian Carcinomas: The Avera Experience

**Figure 2:**
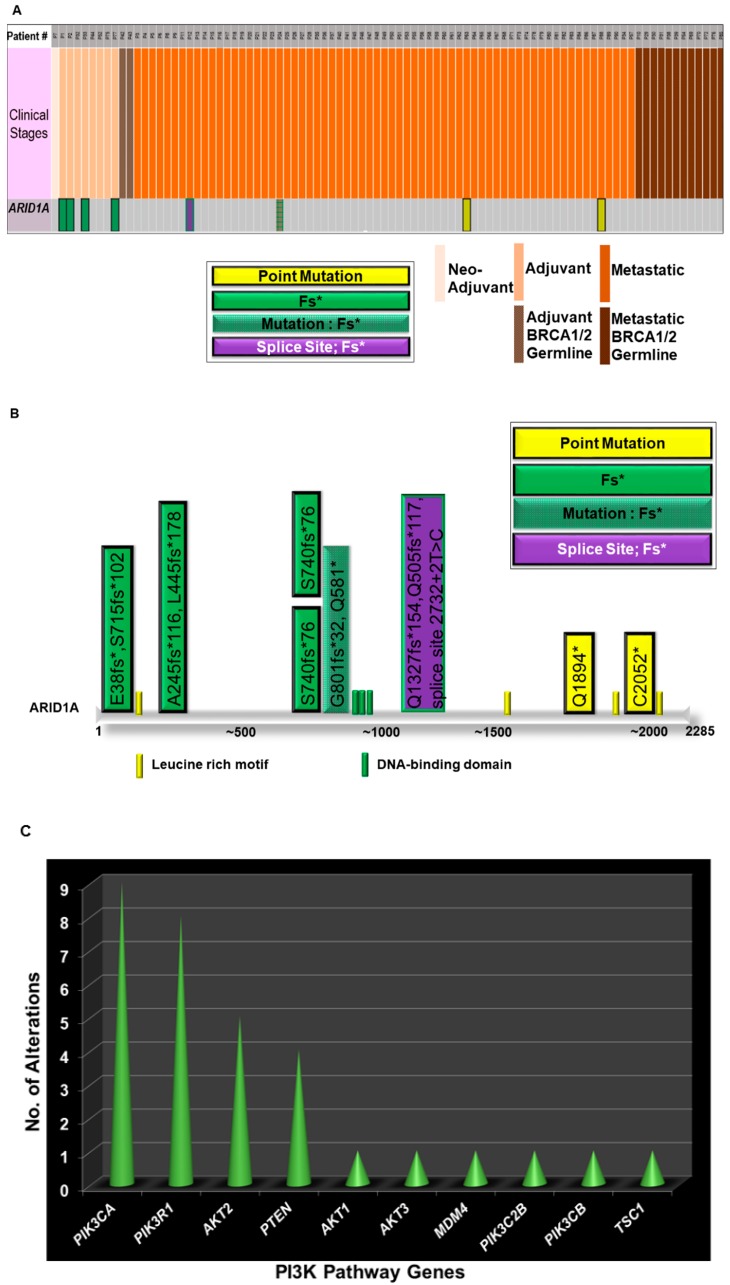

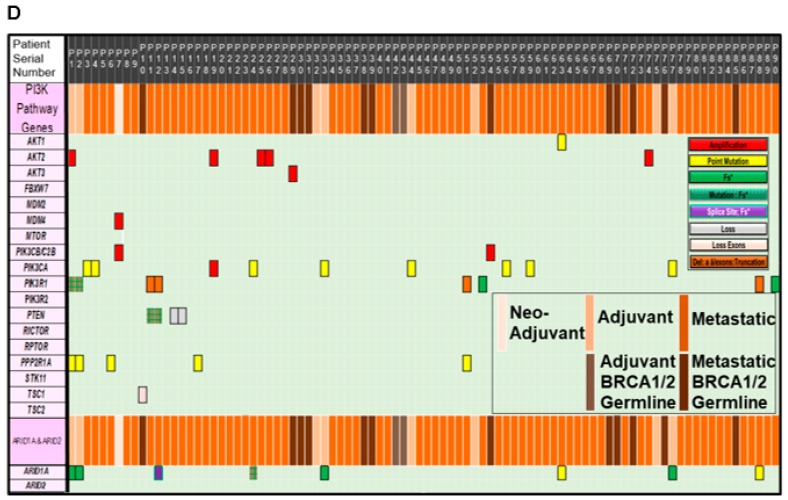
Alterations of ARID1A and genes of the PI3K-pathway genes in Patients with Ovarian Cancers from Avera Cancer Institute. Alterations of ARID1A and genes of the PI3K-pathway as well as a heatmap of co-distribution of these genes in tumors from patients with ovarian cancers at Avera Cancer Institute are presented. De-identified patients were represented with serial numbers. (**A**) presents the spectrum of alteration (number and types of alterations) in the ARID1A gene in tumors from ovarian cancer patients. The data were obtained from FoundationOne reports from 90 ovarian cancer samples. Patients’ tumors were sorted according to the clinical stages, neo-adjuvant, adjuvant, adjuvant BRCA1/2 germline, metastatic, and metastatic BRCA1/2 germline as presented with the color codes as an inset. Different types of alterations recorded in the ARID1A gene are also presented in the figure as an inset. (**B**) presents the mutational spectrum of ARID1A in ovarian cancer cohort of the Avera Cancer Institute. The data were obtained from FoundationOne reports from 90 ovarian cancer samples. Details of the mutations in individual samples are plotted on the linear representation of the ARID1A in the cartoon. Major domains of ARID1A are presented in the cartoon with the approximation of the amino acid numbers. The details of the alteration(s) are color-coded in the figure. Different types of alterations recorded in the ARID1A gene are also presented in the figure as an inset. (**C**) presents the number of alterations recorded in tumors in the genes of the PI3K pathway from patients with ovarian cancers in the Avera Cancer Institute. The data were obtained from FoundationOne reports from 90 ovarian cancer samples. (**D**) presents the co-distribution of alterations (FoundationOne) of the ARID1A gene with the genes of the PI3K pathway as recorded in the 90 ovarian cancer patients at the Avera Cancer Institute. The sorted data has been used to generate a heatmap to present the co-alteration pattern of the types of alterations of ARID1A genes and alterations of the PI3K pathway genes. The vertical column represents a patient, while the horizontal rows represent different genes of the PI3K pathway, ARDI1A gene, and ARID2 gene. The clinical stages, neo-adjuvant, adjuvant, adjuvant BRCA1/2 germline, metastatic, and metastatic BRCA1/2 germline, are presented with the color codes as an inset. Different types of alterations recorded in the ARID1A gene are also presented in the figure as an inset.

We analyzed the expression pattern of different genes in 90 ovarian cancer samples from patients with ovarian cancers whose tumors were subjected to comprehensive genomic-profiling (FoundationOne) who were seen in our Avera Center Institute from February 2014 (Figure 2). This figure is generated out of data from patients from Avera Cancer Institute. Tumors were characterized based on germline status for BRCA1/2, histological types, stages, and specimen sites. We recorded 87% metastatic and 11% adjuvant tumors with a predominant histological type of high grade serous (58%). We observed a total of approximately 318 alterations in 100 genes in 90 ovarian cancer samples. Alterations in TP53 and MYC represented 72% and 25% of tumors, respectively. Within 90 tumor samples, 21% of tumors exhibited an alteration in BRCA1/2 in either somatic or germline (15%). We observed alterations in the ARID1A gene in eight patients whose tumors are predominantly clear cell carcinomas (six out of eight). Out of the eight cases, four were adjuvant, and four were metastatic. Interestingly, none of the patients had alteration in germline BRCA1/2. Four types of alterations were observed, including point mutation, frameshifts, mutation/frameshift, and splice site/frameshift. All frameshift mutations were found in the adjuvant category, while the alterations in the metastatic category were more diverse (Figure 2A). 

As ARID1A is a part of SWI/SNF chromatin remodeling complexes, we also tested genes encoding subunits of this remodeling complex, such as ARID1B, ARID2, SMARCA2, and SMARCA4. As multi-subunit protein complexes, SWI/SNF utilizes the energy of ATP hydrolysis, sliding nucleosomes along a DNA template in the course of remodeling chromatin structure [25]. The SWI/SNF multi-subunit protein consists of (1) the catalytic ATPase subunit (either SMARCA4/BRG1[BRG1)/SMARCA4 (SWI/SNF-related, matrix-associated, actin-dependent regulator of chromatin subfamily A member 4] or SMARCA2/BRM [SWI/SNF-related, matrix-associated, actin-dependent regulator of chromatin subfamily A member 2] [26] and (2) the non-catalytic subunits SMARCB1/SNF5,BAF155, and BAF170 [27]. Among other genes encoding subunits of this remodeling complex, such as ARID1A, ARID1B, ARID2, SMARCA2, and SMARCA4, we observed alterations of SMARCA4 in only 2 out of 90 tumors.

The mutational spectrum of ARID1A alterations is presented in Figure 2B. Details of the mutations in individual samples are plotted on the linear representation of the ARID1A in the cartoon. Major domains of ARID1A are presented in the cartoon with the approximation of the amino acid numbers. In line with the previous report, the alterations were observed along the entire stretch of the gene [15]. We also studied the co-occurrence of ARID1A alterations with alterations of genes of the PI3K pathway in our patients with ovarian cancers. For the purpose, we first identified the distribution pattern of genes of the PI3K pathway in tumors from Avera patients with ovarian cancers, as presented in Figure 2C, which shows the number of alterations recorded in tumors in the genes of the PI3K pathway (Y-axis) from patients with ovarian cancers in the Avera Cancer Institute. The genes of the PI3K pathway included are PIK3CA, PIK3R1, AKT2, PTEN, AKT1, AKT3, MDM4, PIK3C2B, PIK3CB and TSC1. The most number of alterations were observed in PIK3CA, PIK3R1, AKT2, and PTEN genes. The data were obtained from FoundationOne reports from 90 ovarian cancer samples. Finally, we present the co-distribution of alterations of ARID1A genes and alterations of the PI3K pathway genes in tumors from patients with ovarian cancers enrolled at the Avera Cancer Institute. The list of genes of the PI3K pathway was expanded to include STK11, PPP2R1A, and FBXW7. The sorted data has been used to generate a heatmap to present the pattern of alterations of ARID1A genes and alterations of the PI3K pathway genes (FoundationOne). Figure 2D presents the different types of alterations observed in the set of patients with ovarian cancers. The sorted data has been used to generate a heatmap to present the co-alteration pattern of the types of alterations of ARID1A genes and alterations of the PI3K pathway genes. The vertical column represents a patient, while the horizontal rows represent different genes of the PI3K pathway, ARID1A gene, and ARID2 gene. The clinical stages, neo-adjuvant, adjuvant, adjuvant BRCA1/2 germline, metastatic, and metastatic BRCA1/2 germline are presented with the color codes as an inset. Different types of alterations recorded in the ARID1A gene are also presented in the figure as a separate inset. The heatmap showed that all eight patients with alterations in ARID1A had alterations in at least one gene of the PI3K pathway. Most co-alterations included amplification of AKT2, point mutation of AKT1, mutation/frameshift/deletion/truncation in PIK3R1, mutation/frameshift of PTEN, and point mutations of PIK3CA (Figure 2D).

A conditional PTEN deletion within the ovarian surface epithelium has been reported to induce pre-neoplastic ovarian lesions with an endometrioid glandular morphology using the first genetic models of peritoneal endometriosis and endometrioid ovarian adenocarcinoma in mice [28]. Frequent somatic PTEN mutations in endometrioid epithelial ovarian tumors have been reported indicating PTEN’s role in the etiology of this subtype [29]. Interestingly the only patient with mutation/frameshift of PTEN co-occurring with splice site/frameshift alteration in ARID1A had an endometrioid tumor. Genetic mutations in PTEN along with alterations of PIK3CA, PIK3R1, ARID1A, Wnt/β-catenin, microsatellite instability, SRC, and KRAS have been demonstrated to be critical in the pathogenesis of endometriosis-associated ovarian cancers [30,31]. Surprisingly none of our ARID1A alterations in patients with ovarian cancers were companied by high tumor mutation burden (TMB) or microsatellite instability (MSI), albeit our sample number is less. We also observed no alterations in the ARID2 gene, which encodes a member of the SWItch/sucrose non-fermentable chromatin remodeling complex in none of our ovarian tumors. It is understandable as ARID2 mutations are commonly observed in hepatocellular carcinoma and melanoma [32,33,34,35]. 

## 6. ARID1A Protein and its Role in Tumorigenic Transformation

ARID1A protein regulates transcription. Wu and Roberts have presented following modes of action of the SWI/SNF complex to explain its role in the regulation of transcription: (1) mobilizing nucleosomes at promoters, enhancers, and/or gene bodies; (2) facilitating the binding of transcription factors; (3) recruiting coactivator/corepressor complexes; (4) recruiting histone-modifying enzymes; and (5) facilitating chromatin looping to facilitate enhancer and promoter interaction [15]. Thus a transcriptional dysfunction arising from the functional loss of ARID1A, they proposed, would have disruptive effects on the expression or stability of other SWI/SNF subunits, assembly of variant SWI/SNF complexes, the composition or assembly of several SWI/SNF complex variants, its nucleosome sliding activity, targeting to specific genomic loci, and/or recruitment of coactivator/corepressor activities [15]. Located on chromosome 1p, ARID1A is ~250 kD is a widely expressed nuclear protein that is post-translationally modified by N-16-lysine acetylation and serine/threonine phosphorylation [36]. ARID1A and ARID1B are two members of the ARID1 subfamily and are subunits of the SWI/SNF chromatin remodeling complex, which is instrumental in its role as a tumor suppressor as evidenced from the studies understanding how mutation of ARID1A in different organ-type cancers facilitates oncogenic transformation [15,37]. ARID1A and ARID1B are two mutually exclusive subunits of the SWI/SNF chromatin remodeling complex [37]. Although ARID1B has been reported to rescue ARID1A loss mediated transcription by restoring physiological RNAPII activity following its upregulation, multiple p53 and estrogen target genes were found strictly dependent on ARID1A in ovarian clear cell carcinoma [38].

Several studies have identified that ARID1A contributes to tumorigenesis by influencing several cellular functions, (1) proliferation, (2) differentiation, (3) mitosis, (4) DNA damage repair, and (5) apoptosis. *In vitro* knockdown of wt ARID1A/re-expression of clinically relevant mutant ARID1A studies in cancer cell lines and ES cells demonstrated that ARID1A’s effect on the proliferation of normal ovarian surface epithelial cells [39]. Guan et al. reported that restoring wt ARID1A expression in ovarian cancer cells with ARID1A mutations suppressed cell proliferation and tumor growth in mice, whereas RNA interference-mediated silencing of ARID1A in non-transformed epithelial cells enhanced cellular proliferation and tumorigenicity [39]. Guan et al. identified CDKN1A and SMAD3 as downstream targets of ARID1A and showed that wt p53 was required and sufficient for their regulation by ARID1A. Understandably, ARID1A expression is cell-cycle dependent and accumulates in G0 and is downregulated throughout the cell cycle phases but is completely eliminated during mitosis [40]. Growth suppressive effect of ARID1A was mediated by downstream effector of p53, p21 through a direct interaction of the ARID1A/BRG1 complex with p53 and that mutations in the ARID1A and TP53 genes were mutually exclusive in tumor specimens examined [39]. In contrast to their report, we observed the presence of wild type p53 in 50% of the cases with ARID1A alterations. Knockdown of ARID1A inhibited cell cycle arrests [41,42] while in ES cells, BAF250a controlled self-renewal, differentiation, and cell lineage decisions [43]. ARID1A was identified among five regulators of the response to FAS activation in the response of CML cells to imatinib treatment [44]. A completely different mode of action of ARID1A at the promoter level in ovarian clear cell carcinoma that mechanistically regulate ARID1A mediated tumorigenesis has been presented by Trizzino et al. who showed that ARID1A binds most active promoters and enhancers in ovarian clear cell carcinoma and regulates RNA polymerase II promoter-proximal pausing and exclusively contributes to the transcription of multiple p53 and ESR1 target genes [38]. 

By contributing to DNA damage repair and telomere cohesion, ARID1A plays a critical role in maintaining mitotic integrity in a cell. ARID1A promotes STAG1 expression required for telomere cohesion. ARID1A inactivation causes defects in telomere cohesion, leading to DNA damage at telomeres and defects in mitosis. ARID1A inactivation in human ovarian clear cell carcinoma cell line (RMG-I) causes telomere damage that can be rescued by STAG1 expression. Thus ARID1A inactivation is selective against the gross chromosome aberrations and the survival of cells during mitosis [45]. ARID1A recruits MSH2 to chromatin during DNA replication and promotes MMR. ARID1A loss contributes to impaired MMR protein MSH2 and MMR-defective mutator phenotype in cancers [46]. ARID1A deficiency correlated with (1) microsatellite instability genomic signature, (2) a predominant C>T mutation pattern, (3) increased mutagenesis, and (4) increased mutation burden in several cancer types. Interestingly, an increased mutational burden due to a functional loss of ARID1A has been associated with immune phenotypes in tumors, which can be therapeutically exploited by immune checkpoint blockade therapy. Shen et al. reported that tumors formed by an ARID1A-deficient ovarian cancer cell line in syngeneic mice displayed increased mutation load, elevated numbers of tumor-infiltrating lymphocytes, along with PD-L1 expression. Treatment with anti-PD-L1 antibody reduced tumor burden leading to prolonged survival of these mice bearing ARID1A-deficient ovarian tumors as compared to mice bearing ARID1A wild type ovarian tumors [46].

Recruited to DNA double-strand breaks (DSBs) via its interaction with the upstream DNA damage checkpoint kinase ATR, ARID1A impairs DSB-induced ATR activation and regulates the G2/M DNA damage checkpoint by facilitating efficient processing of DSB to single-strand ends, and sustains DNA damage signaling. ARID1A deficiency has been shown to sensitize cancer cells to PARP inhibitor, BMN673 [47]. ARID1A directed lethal strategies can be sought using synthetic lethal targets [48] like DNA repair proteins, including PARP, and ATR, and the epigenetic factors, including EZH2, HDACs, and BRD2. Considering the approval of PARP inhibitors by the FDA [49], novel combinations strategies with PARP inhibitors [50] in ovarian cancer are awaited. Interestingly, a systematic characterization of BAF (subunits of the BRG1/BRM associated factor) mutations opened insights into intra-complex synthetic lethal interactions of explaining intra-complex co-dependencies, including the synthetic lethal interactions of SMARC and ARID components in human cancers [51].

## 7. Co-Alterations of ARID1A with Other Oncogenic Pathways in the Context of Mutation-driven Signaling in Tumor cells

Several potential targets selective against ARID1A mutation in cancers have been proposed based on the context of the cellular signaling in the respective organ-type cancers [14,48,52,53,54]. The most important mode of action is the synthetic lethality of tumor cells in ARID1A mutated cancers with co-occurring mutations of genes pertaining to pathways including the PI3K pathway [55,56,57,58], SRC family kinases [59], DNA-damage repair pathways [60], tumor immune pathway [61], p53 targets/telomerase activation [22,54], Wnt Pathway/PP2A pathway [21,22,62,63], and EZH2 histone methyltransferase as well as BET pathways [48,64,65]. Chandler et al. propose that ARID1A protects against inflammation-driven tumorigenesis in ovarian cancers. Their study established an epistatic relationship between SWI/SNF chromatin remodeling and PI3K pathway mutations in OCCC (ovarian clear cell carcinomas) to demonstrate that a convergence of the pathways on pro-tumorigenic cytokine (IL-6 overproduction) signaling [66]. In endometriosis-associated ovarian cancers with loss of BAF250a, expression of pAKT, γH2AX, BIM, and BAX was higher than in benign endometriosis, whereas expression of pATM, pCHK2, and BCL2 was found lower [67]. ARID1A and ARID1B were localized at the DNA strand breaks to facilitate non-homologous end joining, and ARID1A was assigned a role in homologous recombination [47,68]. Similarly, dysregulation of ARID1A or ARID1B has been shown to sensitize osteosarcoma cells and immortalize pancreatic ductal epithelial cells to cisplatin and oxaloplatin [69]. Furthermore, ARID1A deficient cells acquire high sensitivity to PARP inhibitors following exposure to ionizing radiation [70]. In a mechanistic study, Shen et al. reported ARID1A is required for DNA DSB-induced G2/M arrest, and ARID1A is recruited to DNA breaks via its interaction with ATR via its C-terminal leading to DSB-induced ATR activation. Consequently, ARID1A deficiency sensitizes cells to PARP inhibitors, and PARP inhibitor BMN673 (talazoparib) selectively blocks ARID1A-deficient tumors in xenograft models [47]. The application of the concept of synthetic lethality has initiated several studies combining inhibitors of PARP (olaparib) with PI3K inhibitor (BKM120) [60], testing PI3K-inhibitor LY294002 or AKT-inhibitor MK2206 in ARID1A mutated radio-resistant pancreatic cancer [71]. Recently Kim et al. reported that a loss of ARID1A increased PD-L1 via activating PI3K signaling. Furthermore, they found that microsatellite instability-high tumors had the highest expression of PD-L1 exhibited simultaneous KRAS mutation and loss of ARID1A in gastric cancers [61].

## 8. Is Mutation-Driven Co-Alterations in the ARID1A-PI3K Signaling an Opportunity?

PIK3CA was overexpressed in 73%, while PTEN expression was negative in 5% of the ovarian clear cell carcinomas. Overexpressed PIK3CA, which correlated with the presence of mutation or amplification of the PIK3CA gene in tumors, was found to be associated with p-AKT overexpression [72]. In our cohort, we observed that every patient with alterations in ARID1A had at least one co-alteration of PI3K pathway genes. The predominant genes of the PI3K pathway that was co-altered were PIK3CA (37%) and PIK3R1 (50%), while alteration in PTEN was found in 12% of ARID1A altered tumors. The only type of alteration in the PIK3CA gene was point mutation, while both deletion/truncation and mutation/frameshift were observed in the PIK3R1 gene (Figure 2D). 

Two major genes reported to be mutated in ovarian clear cell carcinoma are PIK3CA and ARID1A, which frequently coexist with each other [73]. A frequent co-occurrence of ARID1A mutations with the alteration of PI3K pathway genes (like PIK3CA encoding the catalytic subunit, p110α) leading to an activation of the pathway has been reported [37,66,74]. The study suggests a cooperating mechanism between the gene-sets. A similar co-occurrence of ARID1A mutations and alterations in the PI3K pathway genes in endometriosis-associated ovarian carcinomas (such as two epithelial ovarian carcinoma subtypes, the ovarian clear cell carcinomas and the endometrioid ovarian carcinomas which have been molecularly and epidemiologically linked to endometriosis) has been observed in our patients. Loss of ARID1A/BAF250a expression led to significantly high levels of AKT-Thr308 and AKT-Ser473 phosphorylation in chemotherapy-naive ovarian carcinomas. In HER2 positive breast cancer model, the loss of ARID1A transcriptionally activates annexinA1 (ANXA1). An elevated annexinA1 protein levels at the plasma membrane activates AKT signaling [75]. Although the causative action of ARID1A has been questioned because siRNA mediated knockdown of BAF250a in OCCC lines (wild type for ARID1A) showed no increase in either pAKT-Thr308 or pAKT-S473 indicating that pAKT in tumor tissues was indirectly regulated by BAF250a expression [58]. In endometrial cancers, ARID1A has been reported as a causative gene of microsatellite instability by having a role in epigenetic silencing of the MLH1 gene [76]. In breast cancer model, a loss of ARID1A expression sensitizes cancer cells to PI3K- and AKT-inhibition and knockdown of ARID1A in MCF7 increased pAKT-Ser473 while treatment with MK-2206 increased apoptosis and decreases pS6K in ARID1A-depleted MCF7 cells. In ovarian clear cell lines ARID1A-deficiency correlated with increased pAKT-Ser473 levels and with sensitivity towards treatment with MK-2206 [57].

The exact cooperative nature of this co-alteration has not been completely understood. In neoplasms originating from the uterine endometrium, a coexistent ARID1A and PI3K mutations promote epithelial trans-differentiation and collective invasion wherein Wilson et al. observed siARID1A and PIK3CA-H1047R convergence on the NFκB pathway, as previously described in ovarian clear cell carcinoma, and the EMT pathway [77]. Interestingly, Zhai et al. observed that ARID1A loss is associated with loss of vimentin expression in human OECs and showed enrichment for genes associated with EMT in the ARID1A-deficient tumors [74]. The extent of oncogenic cooperativity has been tested using bi-allelic inactivation of ARID1A and activation of mutant PIK3CA in the mouse ovarian surface epithelium model. Different studies using both the E545K p110alpha mutation in the helical domain and the H1047R mutation in the kinase domain indicated a domain-specific effect of PIK3CA mutation. In the same context, Chandler et al. demonstrated that bi-allelic inactivation of ARID1A and activation of mutant (H1047R) PIK3CA in the mouse ovarian surface epithelium resulted in a highly penetrant form of ovarian clear cell carcinoma [66]. Studies by Zhai et al. have shown that PIK3CA mutations affecting the kinase and helical domains in the context of conditional ARID1A inactivation have different functional consequences of tumor development and the neoplastic potency in mouse ovarian surface epithelium [74]. One of the modes of cooperation of the PI3K/AKT pathway and ARID1A deficiency involves inflammatory cytokine signaling, and coexistent ARID1A-PIK3CA mutations have been reported to promote ovarian clear-cell tumorigenesis through sustained pro-tumorigenic IL-6 overproduction [66].

Collectively, studies so far strongly suggest a cooperating mechanism between the two co-occurring ARID1A and PI3K pathways [15,37]. It is possible that during the course of neoplastic transformation, a mutation in the ARID1A gene and genes of PI3K pathway develop oncogenic cooperativity (a specific requirement of the PI3K/AKT pathway in ARID1A-deficient tumors). Interestingly such a situation creates the best scenario for a synthetic lethal interaction between the loss of ARID1A expression and inhibition of the PI3K/AKT pathway in tumor cells. Inhibition of PI3K/AKT signals has been shown to radio-sensitizes pancreatic cancer cells with ARID1A deficiency *in vitro* [71].

## 9. Epilogue

**Figure 3:**
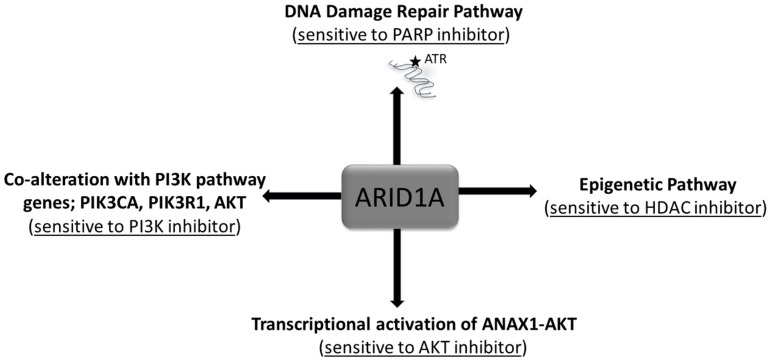
Alteration of ARID1A and treatment options. The alterations of ARID1A has been presented along with the various treatment options from the viewpoint of tumor cell signaling.

The most commonly encountered mutations of ARID1A lead to a loss of expression/function of ARID1A, making ARID1A a poor therapeutic target. Hence studies are conducted to find out the molecular consequences of ARID1A deficiency to identify therapeutic options in ARID1A-mutant tumors. The dependence on HDAC6 activity in ARID1A-mutated ovarian cancer cells has been reported to be correlated with direct transcriptional repression of HDAC6 by ARID1A as HDAC6 inhibition selectively promoted apoptosis of ARID1A-mutated cells [78]. As a pro-apoptotic post-translational modification, HDAC6 directly deacetylates Lys120 of wild type p53. Thus, ARID1A mutation inactivates the apoptosis-promoting function of p53 by upregulating HDAC6. ARID1A loss of function has been prevalently recorded to occur concurrently with alteration of genes of other oncogenic pathways. Being a tumor suppressor orchestrating transcription via nucleosome remodeling, the clinical evaluation of the degree and depth of loss of ARID1A remains to be completed in cancers. Figure 3 summarizes alterations of ARID1A and treatment options from the viewpoint of tumor cell signaling. As we know more about the functional cooperativity of the co-alterations occurring with the loss of function of ARID1A, our approach to managing the progression of a disease and the resistance to treatment will be refined. 

It remains to find out the role ARID1A plays in the continuum model for tumor suppression [79] and how a loss of ARID1A function cooperates with either the concurrent or sequential activation of major oncogenic pathways like the PI3K and RAS-MAPK pathways, epigenetic pathway and DNA damage repair pathway.
